# Functionalization of TiO_2_ and Janus-TiO_2_ Nanoparticles with Organosilanes for Tunable Pickering Emulsification and Photocatalytic Wastewater Treatment

**DOI:** 10.1021/acsomega.5c05844

**Published:** 2025-09-23

**Authors:** Zygimantas Gricius, Gisle Øye

**Affiliations:** Ugelstad Laboratory, Department of Chemical Engineering, 8018 Norwegian University of Science and Technology (NTNU), Trondheim 7491, Norway

## Abstract

This study examines the use of organosilanes to functionalize titanium dioxide (TiO_2_) and Janus-TiO_2_ nanoparticles, enabling precise control of surface chemistry for stabilizing Pickering emulsions. Organosilanes with varying hydrophobic chain lengths—3-aminopropyltrimethoxysilane (APS), octyltrichlorosilane (OTS), and trichloro­(octadecyl)­silane (ODTS)—were employed to modify TiO_2_ surfaces, creating nanoparticles with tailored wettability. Comprehensive characterization, including contact angle measurements, FT-IR, TGA, and zeta potential analysis, confirmed successful modification and demonstrated the correlation between nanoparticle surface properties and emulsification efficiency. APS-functionalized particles exhibited enhanced emulsion stability, achieving prolonged stability through droplet flocculation and reduced mobility. An optimal contact angle window (∼10°–40°) was identified for effective emulsification, providing a framework for designing robust Pickering emulsifiers. Comparative analysis revealed ODTS’s superior performance over OTS, attributed to its longer alkyl chain imparting steric hindrance, which was insufficient in OTS-modified particles. Photodegradation studies of a model naphthenic acid revealed enhanced emulsion stability for Janus-TiO_2_ and TiO_2_-APS emulsions, enabling easy photocatalyst reuse and recovery. These findings offer valuable insights for the development of versatile, surface-engineered Pickering emulsifiers with potential applications in photocatalytic wastewater treatment applications and beyond.

## Graphical abstract

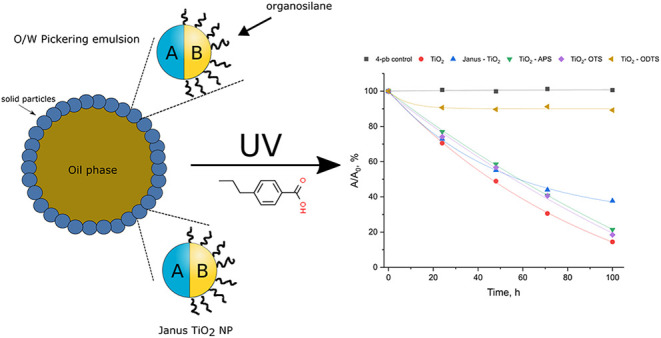

## 1 Introduction

Pickering emulsions (PE) represent a unique class of emulsions stabilized by solid particles adsorbed at the liquid–liquid interface.^1^ In recent years, such emulsions have gained significant attention due to their enhanced stability, reusability, and applications in areas like food science, wastewater treatment, drug delivery, and advanced materials.^2–5^ Their enhanced stability originates from the adsorbed particles, which form a physical barrier that prevents coalescence of the dispersed droplets.^6^ The extent of particle adsorption at the oil–water interface depends on the wettability of the particles, which determines their ability to remain anchored at the interface.^7,8^ Therefore, by adjusting the surface chemistry of the particles, such as through functionalization or anisotropic modifications, the stability of Pickering emulsions can be finely tuned.^8,9^

Titania nanoparticles (TiO_2_ NPs) have been used as emulsion stabilizers in various applications due to their high surface area, photocatalytic efficacy, environmental stability, and versatility in functionalization.^10–12^ However, their inherent hydrophilicity limits their efficiency as emulsifiers, necessitating surface modification to enhance interfacial adsorption.^8^ Functionalizing TiO_2_ with wetting agents provides means to tailor surface properties, enabling selective wettability and interfacial activity. For example, fluorination of TiO_2_ facilitated the stabilization of emulsions with hydrophobic pollutants like nitrobenzene, thereby improving photocatalytic degradation rates.^11^ Similarly, improved photodegradation of chlorobenzene was observed when salicylic acid-modified titania particles were used as Pickering emulsifiers.^13^

Over the past decades, the increasing use of nanomaterials and nanocomposites has sparked interest in using organosilanes as surface modifiers, as many applications demand nanoparticles that are chemically stable, uniformly sized, and effectively dispersed within their respective media.^14–16^ Organosilanes can be described by the formula R­(4 – x)­Si­(OR′)_*x*_, where *x* ranges from 1 to 3.^16,17^ Here, OR′ represents a hydrolyzable alkoxy group, and R is an organic functional group that gives rise to the desired surface properties. The modification process consists of two main steps: initially, silanes hydrolyze to produce silanols, which subsequently undergo a condensation reaction with hydroxyl groups on the substrate surface. In the process, covalent bonds between the surface and the organosilanes are formed, exposing organic functional groups at the particle surface.

Despite the extensive research on the surface modification of metal-oxide nanoparticles with silane coupling agents, several knowledge gaps remain, particularly in the context of their application in Pickering emulsions. First, many studies focus predominantly on enhancing the general dispersibility or stability of titania dispersions in aqueous or organic media, often neglecting the specific interfacial behavior required for stabilizing emulsions.^14,17^ The unique requirement of intermediate wettability necessary for efficient Pickering emulsification is seldom systematically explored. Another route of surface modification for Pickering emulsion stabilization relies on producing Janus particles, characterized by their dual surface chemistry with distinct hydrophilic and hydrophobic regions.^18–20^ Such particles are uniquely suited for stabilizing Pickering emulsions due to their asymmetric wettability.^21^ However, to the best of our knowledge, no studies on organosilane-modified Janus-TiO_2_ Pickering emulsions used in photocatalytic wastewater treatment have been reported. This route offers a rather simplistic approach to produce amphiphilic particles capable of stabilizing Pickering emulsions much more effectively than the conventional TiO_2_, a property that is critical for enhancing the recyclability of photocatalytic systems. The emulsion stability during the photodegradation process is yet another area that has not been explored in the literature.

To address these gaps, the work focuses on four key aspects: (i) optimizing the Janus-TiO_2_ preparation method to ensure uniform adsorption of titania particles at the wax–water interface; (ii) synthesizing Janus-TiO_2_ particles smaller than 1 μm to enhance surface area for photocatalytic reactions; (iii) establishing a relationship between organosilane structure and emulsion properties; and (iv) analyzing the impact of functionalization on the emulsion stability and photodegradation kinetics of model pollutants.

The first two goals were pursued through modification of TiO_2_ with CTAB to promote particle adsorption at the liquid wax/water interface. The emulsion properties were investigated by grafting three organosilanes—3-aminopropyltrimethoxysilane (APS), octyltrichlorosilane (OTS), and trichloro­(octadecyl)­silane (ODTS)—onto both the TiO_2_ and Janus-TiO_2_ nanoparticles. The silanes were chosen to systematically vary the surface properties of TiO_2_, offering a gradient of hydrophobicity for Pickering emulsification. Through this process, APS introduced amine groups, while OTS and ODTS progressively increased particle hydrophobicity through alkyl chains of differing lengths. The diverse selection of the silanes allowed for the tuning of TiO_2_’s wettability and interfacial activity, enabling precise control over the stabilization of oil-in-water emulsions. Finally, the performance in photocatalytic breakdown of 4-propylbenzoic acid (4-pb) was assessed for the most interesting PE systems.

## 2 Experimental Section

### 2.1 Materials

All samples were prepared using Milli-Q water with a resistivity of 18.2 MΩ/cm at 25 °C from the Millipore water system (Darmstadt, Germany). Titanium­(IV) oxide nanopowder (TiO_2_ NP, ≥99.5%), 4-propylbenzoic acid (4-pb), 3-aminopropyltrimethoxysilane (APS, 97%), octyltrichlorosilane (OTS, 97%), trichloro­(octadecyl)­silane (≥90%), and silicone oil (viscosity 5 cSt, 25 °C), were purchased from Sigma-Aldrich (Schnelldorf, Germany). Cetyltrimethylammonium bromide (CTAB, 99%+) was purchased from Acros Organics. Sasolwax 5405 was obtained from Sasol Wax GmbH. DCM (ACS reagent, ≥99.9%), acetone (ACS reagent, ≥99.5%), ethyl acetate (ACS reagent, ≥99.5) and chloroform (HPLC, grade, ≥99.8%) were purchased from Sigma-Aldrich (Schnelldorf, Germany). Methanol (ACS reagent) was acquired from VWR International (Darmstadt, Germany). All chemicals were used as received.

### 2.2 TiO_2_ NP Etching

An etching step was introduced to improve functionalization efficiency by removing surface contaminants, which resulted in a higher exposure of Ti–OH groups on the particle surface. One g commercial 21 nm TiO_2_ powder was placed into a tall beaker, containing 20 mL concentrated sulfuric acid under stirring at 300 rpm. Upon gradually adding 8 mL of 30% H_2_O_2_, the resulting dispersion became pale yellow. The open mixture was left at ambient temperature for 2.5 h to allow peroxide decomposition. Afterward, the contents were neutralized with NaOH until the pH reached 7, a step performed cautiously in an ice bath due to its exothermic nature. The neutralized dispersion was filtered through a 0.22 μm filter, followed by extensive washing with MQ water. The collected powder was dried overnight at 60 °C in a drying oven. This treatment did not affect the dispersibility, size, or morphology of the particles, as confirmed by separate tests and scanning electron microscopy imaging. Etched titania was used as the base material throughout the study.

### 2.3 Organosilane Functionalization of TiO_2_ NPs

To obtain TiO_2_–OTS and TiO_2_–ODTS, 0.4 g of the etched-TiO_2_ was dispersed in 40 mL of DCM under stirring. Then, 2.5 mM of OTS/ODTS was introduced, and the mixture was stirred for 2 h at room temperature (RT), during which the particles became well-dispersed. The modified TiO_2_ was filtered through a 0.22 μm filter, washed extensively with DCM, and left to dry overnight at room temperature inside a fume hood. The dried powder was ground with a mortar and pestle and stored in a vial. For the TiO_2_-APS, an additional prehydrolysis step was introduced to activate the organosilane. 1 M solution of APS in the methanol/water (80:20) mixture was prepared and hydrolysis was carried out at RT for 3 h, following the work by Salon et al.^22^ Then, the prehydrolyzed APS solution was diluted to obtain selected concentrations (1, 10, 100, and 1000 mM) and added to 20 mg/mL TiO_2_ dispersions stabilized at pH 3 at 1:1 (v/v) ratio. Each mixture was stirred for 3 h at room temperature inside a fume hood. The resulting TiO_2_-APS particles were filtered through a 0.22 μm filter, thoroughly washed with methanol, and left to dry overnight at RT inside a fume hood.

### 2.4 Preparation of Janus-TiO_2_ Particles

0.4 g of etched titania nanoparticles were dispersed in 20 mL pH 3 HCl solution. The nanoparticles were flocculated in a dried state, therefore, an ultrasonic homogenizer (Fisherbrand Model 505, 2 min, 2 s pulse mode, 40% amplitude) was used to produce stable dispersions of clustered titania around 130 nm in diameter, confirmed by dynamic light scattering (DLS) experiments.

For the TiO_2_–CTAB particles, selected concentrations of CTAB were attained by adding a calculated volume of 100 mM CTAB stock solution to TiO_2_ dispersions. The dispersions with CTAB were left overnight under stirring at room temperature in order to reach the adsorption equilibrium. Next day, the particles were washed 3 times by centrifugation and redispersion in pH 3 HCl solution. Successful adsorption of CTAB was confirmed by carrying out zeta potential measurements, which indicated a positive shift in the values for the samples, subjected to CTAB.

For the Pickering wax synthesis, 1 g of Sasolwax 5405 wax was weighted and subjected to 75 °C in an oil bath to completely melt the wax. Another vial containing TiO_2_ dispersion at pH 3 was placed in the same bath for 30 min to fully equilibrate with the temperature. Afterward, both vials were mixed and emulsified for 1 h at 10 000 rpm using an Ultra Turrax T25 homogenizer with an S25 N-8 G dispersing tool, according to the modified procedure by Hong et al.^23^ The resulting wax-titania Pickering emulsions were imaged by optical microscopy to verify the formation of a titania film on the wax droplet surface (Figure S1). Next, the waxes were cooled to room temperature, resulting in the solidification of the wax. The solid wax-titania particles were filtered and washed multiple times with pH 3 HCl solution and deionized water to remove free and weakly attached particles. Finally, the washed wax particles were placed on a laboratory bench for drying at ambient temperature.

For the Janus particle functionalization, the exposed surfaces of the solid wax-titania particles were dispersed in a 2.5 mM solution of OTS/ODTS in DCM for 2 h. The reaction mixture was later rinsed with DCM to eliminate excess silane. To retrieve the modified titania particles, the wax particles were dissolved in chloroform at 45 °C. The dispersion containing dissolved wax was filtered and washed with chloroform and DCM repeating the procedure used for the isotropically coated particles. No wax was visually observed after drying the following day.

### 2.5 Particle and Emulsion Characterization

The particle sizes and zeta potentials were determined using a Malvern Zetasizer Nano-ZS, with a 2 min temperature equilibration period before the measurements were initiated. The pH of the solutions was varied from 3 to 10 by using different buffers (acetate, phosphate, or borax) depending on the target pH value with small adjustments by either 0.1 M NaOH or 0.1 M HCl. The imaging of the produced emulsions was done by optical microscopy (Nikon Eclipse ME600). The droplet size distributions of the produced and photodegraded emulsions were characterized by laser diffraction using a Mastersizer 3000 (Malvern Instruments). Fourier Transform Infrared Spectroscopy (FT-IR) measurements were carried out using a Thermo Fisher Scientific Nicolet iS50 FT-IR spectrometer equipped with an attenuated total reflectance (ATR) accessory. All the readings were done with the same parameters: the resolution of 4 cm^–1^ and 64 scans per measurement. Contact angles were measured using a Teclis Tracker Drop tensiometer (Teclis Scientific) with a 2 μL MQ water droplet on thin films of silane-modified titania. These films were prepared by spin-coating titania dispersions onto microscope glass slides by using a POLOS SPIN150i instrument. Organosilane coating efficiency was analyzed using a thermogravimetric analyzer (NETZSCH, TG 209 F1 Libra). Approximately 10 ± 0.5 mg of sample was placed in a crucible for each experiment and heated from 25 to 600 °C at a rate of 10 °C/min, under nitrogen gas at a flow rate of 40 mL/min. Calcination was performed by heating the samples to 600 °C at a rate of 3 °C/min and maintaining this temperature for 2 h. The Hitachi TM3030 SEM Tabletop Microscope was used to inspect the morphology of the TiO_2_-wax particles. Fifteen kV current and a mixed secondary/backscattered electron imaging mode was used.

### 2.6 Emulsion Mixing

Pickering emulsion samples, each with a total mass of 20 g, were prepared by diluting 2 mg/mL silane-TiO_2_ dispersions at either pH 3 or 11 and adding the silicone oil to achieve a final particle concentration of 1 mg/g. The silicone oil content was maintained at 10 wt % throughout testing. The emulsions were formed by mixing the samples for 2 min at 20,000 rpm. The resulting emulsions were confirmed to be oil-in-water types through a drop test, as the emulsions dispersed uniformly when diluted in an aqueous phase. Accelerating aging was done by subjecting 20 g emulsions to centrifugation at 5000 rpm for 1 min (Thermo Scientific Heraeus Multifuge X3 Centrifuge). The free oil was collected from the top of each sample, weighted and compared to the mass originally added into the mixture.

### 2.7 Photocatalytic Testing

One mg/g emulsions mixed at pH 11 were used to prepare test samples for the photocatalytic degradation studies. 500 μL from each emulsion were added to 10 mL 5 mM borax buffer, containing 3 mM 4-pb pollutant. The samples were placed in a UV chamber under continuous stirring, exposed to a light intensity of 4000 μW/cm^2^ for 100 h. At specific time intervals, 1 mL aliquots were taken, centrifuged, and filtered using a 0.22 μm cellulose acetate filter. Then, 300 μL of the supernatant was collected and diluted with the borax buffer 10-fold in a quartz cuvette. The absorbance was measured using a Cary 3500 Multicell UV–vis spectrophotometer (Agilent), capturing spectra within the 200–800 nm range. The absorbance at 235 nm was used to assess the degradation of 4-pb.

## 3 Results and Discussion

### 3.1 Janus Particle Preparation

The method to obtain Janus particles relied on the formation of particle-stabilized wax emulsions, which underwent a limited coalescence process.^23,24^ During mixing, energy was provided to create oil–water interfaces in a form of molten wax droplets, coated by the adsorbed particles. However, depending on the wettability of the particles, the extent of adsorption to the oil–water interface varied. As a result, when stirring was stopped, the partially unprotected droplets coalesced. The process continued until the interface was sufficiently stabilized by the particles, ultimately producing the final emulsions.

Based on the literature, a higher ratio of particles-to-wax was shown to result in smaller emulsion droplet sizes.^24^ However, for hydrophilic particles, such as silica or titania, only a small fraction of particles becomes immobilized at the oil–water interface rendering the synthetic approach of Janus particles inefficient. Another way to approach this limitation is by modifying the surface chemistry of the particles to promote their adsorption to the oil–water interface. That way, the dispersed particles would be better utilized making the synthesis of Janus particles more cost-effective for potential upscaling. Given that titania nanoparticles were intrinsically hydrophilic, it was necessary to partially hydrophobize their surfaces to facilitate the adsorption at the oil–water interface.

To increase the hydrophobicity of particles in the wax-in-water emulsion, we studied the influence of CTAB surfactant by varying its concentration in the TiO_2_ dispersions prior to emulsification. Upon cooling, the emulsions solidified producing wax particles, coated by titania (Figure 1b). However, both the amount of wax added and the extent of surface modification by CTAB had to be optimized for.

**Figure 1:**
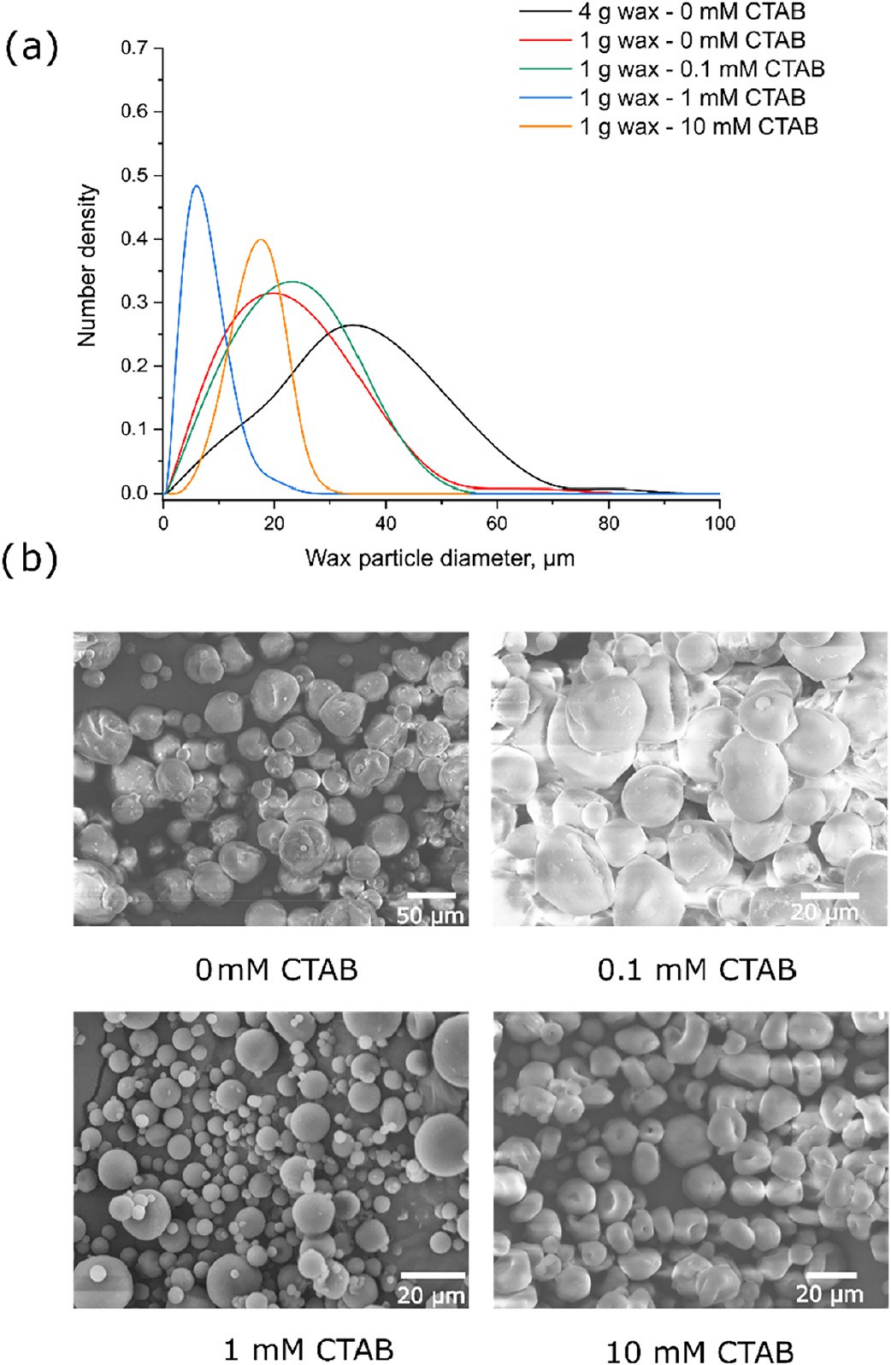
Number distributions of selected Pickering wax particles (a), low magnification SEM images of Pickering wax particles, stabilized by CTAB—functionalized titania nanoparticles, 1 g wax used in all conditions (b).

Figure 1a depicts particle size distributions of wax particles prepared by different amounts of wax and CTAB in titania modification. In the absence of CTAB, the particle size distributions were found to be generally broader, indicating insufficient and varied wax coverage by titania NPs. This hypothesis was supported by the reduction in the peak particle size and the polydispersity observed for the 1g wax system, which effectively increased the particle-to-wax ratio. As a result, a higher number of particles became available to achieve the surface coverage needed to prevent emulsion droplet coalescence. However, no major difference in size and polydispersity was found at lower amounts of wax among the tested samples, suggesting an optimal particle-to-wax mass ratio revolving around 0.4. Still, the unmodified titania-stabilized waxes exhibited a relatively broad size distribution with most of the particles left out in the water phase. To promote the adsorption to the wax-water interface, the influence of CTAB on titania was analyzed. While there was little to no change for the 0.1 mM CTAB formulation, when the concentration was raised to 1 mM, a significant reduction in the wax particle size (5.2 μm) was observed. The difference in size is well illustrated by analyzing the SEM images, shown in Figure 1b, where both the 1 and 10 mM CTAB samples exhibited smaller particles compared to 0 and 0.1 mM CTAB formulations. However, when comparing the 1 and 10 mM CTAB formulations, the 10 mM sample exhibited a higher average particle size (14.1 μm), but much lower PDI (0.07 versus 0.38) values. Furthermore, the morphology of the wax particles changed from spherical to disk-shaped. This behavior suggested a potential leaching of CTAB from the surface of titania resulting in a synergetic stabilization by both titania and CTAB. This was justified by the fact that wax droplets undergo shrinkage upon cooling. However, the rigid CTAB-titania layer exhibits a higher mechanical resistance to deformation compared to the surface, stabilized by CTAB. Consequently, the system minimizes its free energy by deforming the droplets into compressed discs to accommodate the internal shrinkage. It is important to note that CTAB alone could not stabilize wax emulsions possibly because molecular surfactants lacked the surface rigidity provided by particles, which was necessary to physically prevent droplet merging. Therefore, the increase in the particle size observed between 1 and 10 mM CTAB could be attributed to the limited coalescence phenomenon, justified by the fact that TiO_2_–CTAB particles competed with the free CTAB molecules for adsorption at the wax-water interface.

#### 3.1.1 Surface Morphology

Higher magnification SEM images of the produced wax particles were taken to assess the effect of CTAB on the surface coverage and morphology (Figure 2). Without CTAB (0 mM), titania adsorbed on the wax surface in aggregated clusters characterized by irregularly coated multilayers, prone to droplet coalescence. At 0.1 mM CTAB, the surface coverage by TiO_2_ appeared visually smoother, though minor clusters remained. At 1 mM, the wax particles appeared highly uniform, spherical, and smooth, indicating uniform stabilization and minimal coalescence. Similar smoothness was obtained for the 10 mM case with the main difference being the deformation and compression of droplets into disc-like shapes. Due to these deformations, some clustered particles were found to be trapped in the valleys of the waxes. To test the extreme case of CTAB adsorption onto titania, the pH was raised from 3 to 11, which resulted in electrostatically driven adsorption of CTAB on titania resulting in highly hydrophobic particles, which immediately aggregated and adsorbed on the wax surface as dense multilayer clusters, which were not suitable for the controlled Janus particle preparation (Figure S2). This progression highlighted the balance between CTAB concentration used for the TiO_2_ NP functionalization, wax surface stabilization, and mechanical resistance. Given the higher wax particle size and the excess CTAB present on titania, the 10 mM formulation was considered less attractive than the 1 mM system, which was ultimately adopted as the optimal configuration for the Janus particle preparation. Next, the optimized Pickering waxes were surface modified by organosilanes to alter the wettability of the surfaces that were not attached to the waxes.

**Figure 2:**
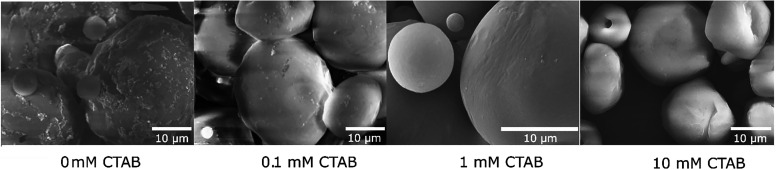
Higher magnification SEM images of Pickering wax particles, stabilized by CTAB—functionalized titania nanoparticles; 1 g wax samples.

### 3.2 FT-IR Analysis

To investigate the outcome of surface modifications of TiO_2_ and Janus-TiO_2_ particles, the FT-IR analysis was performed. The analysis aimed to confirm the functionalization of TiO_2_ with APS, OTS, ODTS, and Janus configurations by identifying the characteristic functional groups introduced during the process. The spectra were compared to assess the impact of different functionalization approaches on the surface chemistry of the particles. APS-functionalization of titania was seen as a route to hydrophobize TiO_2_ in a gentle manner due to the presence of alkyl groups, which could partially mask the surface hydroxyl groups. However, it would still maintain hydrophilicity due to the polar amine groups, making the particles only slightly more hydrophobic for stabilizing Pickering emulsions. In contrast, the hydrophobic silanes such as OTS and ODTS were selected as more extreme wettability agents to investigate the differences in Pickering emulsion stabilization between the Janus particles and isotropically coated titania.

In Figure 3a, the FT-IR spectra of TiO_2_ particles incubated in varying concentrations of APS solutions are shown. While there was no difference observed between the 0 and 10 mM APS samples, distinct new peaks were noted for the 100 and 1000 mM formulations. Characteristic peaks for N–H stretching (∼3300–3500 cm^–1^) and N–H bending (∼1600 cm^–1^) were observed, along with C–H stretching (∼2920 and 2850 cm^–1^) from the methylene groups. Since the amino groups were introduced at the expense of the surface-hydroxyl groups, a substantial decrease and shift in the broad hydroxyl peak intensity at ∼3340 cm^–1^ was also noted. Most notably, the two new peaks at ∼1013 and ∼1102 cm^–1^ were attributed to the Si–O–Ti and Si–O–Si bond vibrations confirming the grafting of APS onto the TiO_2_ surface. While the Si–O–Ti vibration represented the directly grafted organosilanes, the Si–O–Si bonds could have originated from the cross-linking reactions between the neighboring silanes at the surface of titania as previously shown for silicon surfaces.^25^

**Figure 3:**
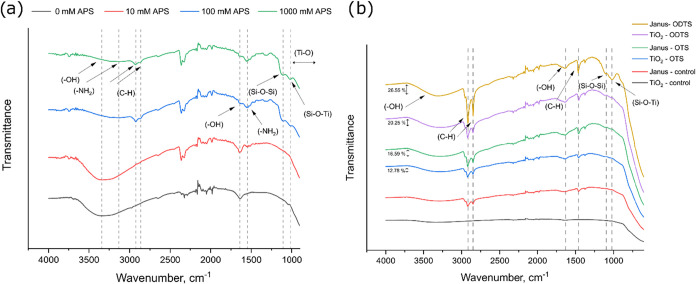
FT-IR analysis of TiO_2_ – APS particles (a), OTS/ODTS functionalized TiO_2_ and Janus-TiO_2_ particles (b).

Similar to APS, the FT-IR spectra of OTS/ODTS-functionalized TiO_2_ and Janus-TiO_2_ particles showed peaks corresponding to C–H stretching (∼2920 and 2850 cm^–1^) and C–H bending (∼1460–1400 cm^–1^), confirming the presence of long alkyl chains (Figure 3b). The Si–O–Ti bond at ∼1025 cm^–1^ was once again detected, suggesting successful silane grafting. The spectra of Janus-TiO_2_ particles resembled that of OTS/ODTS-functionalized TiO_2_, suggesting effective functionalization. Notably, the peak intensities in the alkyl region were higher for the Janus particles compared to the isotropically coated titania. This suggested a greater surface density of the alkyl chains on the Janus particles, likely due to the reduced surface area available for functionalization, which enhanced the signal contribution from the alkyl groups. What is more, higher peak intensities were noted in the hydroxyl region (∼3000–3500 cm^–1^) for the Janus particles, indicating dual functionalization. Some alkyl chain groups were also discovered in the Janus-control sample most likely originating from CTAB or wax residues that could not be fully removed during washing. Finally, the control TiO_2_ lacked both C–H and Si–O peaks, confirming the absence of alkyl chains. While our results are consistent with the formation of Janus TiO_2_–silane nanoparticles, more advanced techniques (e.g., HAADF-STEM coupled with EDX) could provide direct evidence of the hemispherical distribution of silane on the nanoparticle surface.

### 3.3 Thermogravimetric Analysis (TGA)

To supplement the findings of the FT-IR studies, the TGA analysis was conducted as it would provide quantitative information on the extent to which the titania particles have been functionalized by different organosilanes. Figure 4 a depicts the mass change from the APS-functionalized titania. For both the untreated TiO_2_ (0 mM APS) and 10 mM APS samples, the weight loss was minimal (1–1.5%) across the temperature range, likely due to the loss of surface-bound water. In contrast, for the 100 and 1000 mM APS-treated particles, weight loss revolved around 3% at 600 °C, suggesting the decomposition of APS molecules grafted onto the TiO_2_ surface. This pattern matched the FT-IR results, which showed the APS-characteristic peaks only at 100 and 1000 mM APS concentrations. Regarding the thermal degradation pattern, two distinct weight loss regions were identified. The temperature range of 100–300 °C was attributed to the loss of physically adsorbed water, whereas in the range of 300–600 °C, the major weight loss was attributed to the breakdown of the alkyl and amine groups grafted onto the TiO_2_ surface. Overall, the weight loss became significant only at high APS concentrations, reflected by a higher slope in mass loss due to the surface coverage of APS molecules.

**Figure 4:**
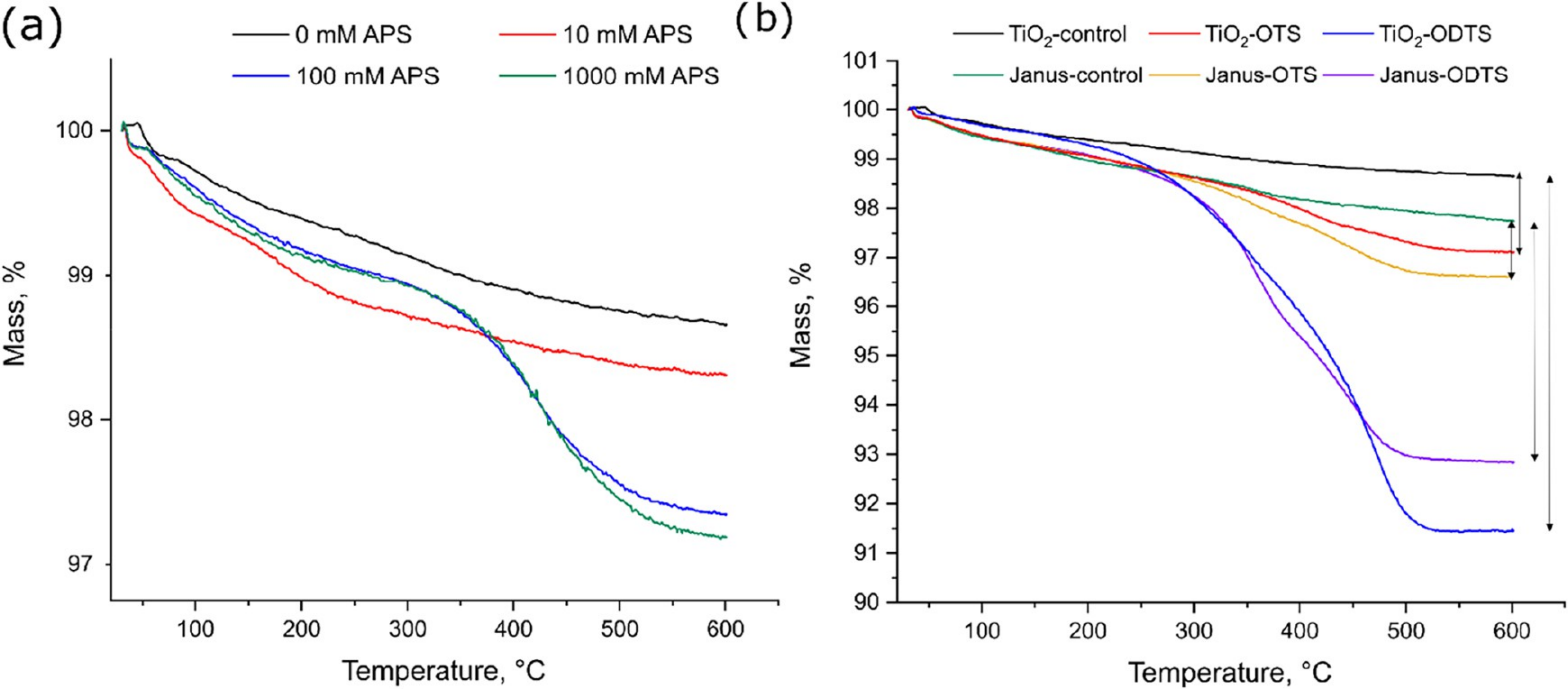
TGA analysis of TiO_2_ – APS particles (a), OTS/ODTS functionalized TiO_2_ and Janus-TiO_2_ particles (b).

Similarly, Figure 4b compares the thermal degradation of both isotropically coated and Janus-TiO_2_ particles, modified by the hydrophobic organosilanes. More significant differences in mass loss were observed, particularly for the ODTS-functionalized particles (∼8.5%). This result was anticipated, as the thermal degradation of organosilanes is primarily driven by the decomposition of the long alkyl chains attached to the surface. Since ODTS possesses an octadecyl hydrocarbon chain, compared to the octyl chain in OTS, a higher degree of degradation per grafted molecule was expected. Furthermore, since both silanes were grafted to titania at the same concentration (2.5 mM) and had the same trichloro leaving groups, comparable adsorbed amount was expected. That was different in the case of APS, where the methoxy groups were known to be worse leaving groups compared to the chloro groups. Regarding the differences between the Janus and isotropic particles, lower mass change was observed for the Janus particles arising from their asymmetric surface coverage, where only part of the surface was available for functionalization. However, since the Janus-control sample exhibited a higher mass loss compared to the TiO_2_-control, the Janus-OTS sample showed a higher absolute mass loss, surpassing the TiO_2_–OTS. The higher mass loss of the Janus-control powders was attributed to the incomplete removal of waxes and CTAB during the preparation of Pickering waxes. Therefore, the influence of the control samples had to be taken into account to isolate the mass loss due to functionalization by the organosilanes. The mass changes of the tested samples, adjusted for their respective control sample degradation values, were summarized in Table 1.

**Table 1:** Change in Sample Mass Calculated from TGA and Calcination Experiments

formulation	TGA, mass change^a^ %	calcination, mass change^a^ %
TiO_2_-APS-10 mM	0.36	1.85
TiO_2_-APS-100 mM	1.31	2.52
TiO_2_-APS-1000 mM	1.47	4.42
TiO_2_–OTS	1.53	9.49
TiO_2_–ODTS	7.20	14.07
Janus-OTS	1.09	7.74
Janus-ODTS	4.90	11.65

a Control sample values subtracted.

Comparable trends in the values were also obtained when the mass of the powders was recorded before and after the calcination at 600 °C in air. During the TGA analysis under the flow of nitrogen, organic groups tend to decompose via pyrolysis rather than combustion. This process left behind a residue of elemental carbon as a byproduct of incomplete decomposition. In contrast, clean TiO_2_ powders were obtained when the samples were calcined. When comparing the values between the two methods, equivalent degradation patterns were observed, with the Janus particles exhibiting lower mass loss compared to the isotropically coated particles.

### 3.4 Contact Angle Measurements

The outcome of surface modification of titania was assessed by measuring contact angles of water droplets on thin films, prepared by spin-coating the functionalized titania dispersions (Figure 5a). The figure shows the variation in the contact angle values for different TiO_2_ samples, comparing the isotropically coated particles with the Janus particles. At first, clean glass slides were tested as control samples exhibiting values around 18°. Next, the TiO_2_-control and TiO_2_-APS (100 mM) were identified as the most hydrophilic surfaces with the contact angle around 10°. While the observed hydrophilicity was anticipated for the etched TiO_2_, similar behavior of the TiO_2_-APS samples was rather surprising. However, accurate measurements of contact angles below 10° were difficult to achieve as the liquid droplet spread almost completely across the surface. In contrast, the TiO_2_–OTS and TiO_2_–ODTS exhibited substantially higher contact angles (∼62° for OTS and ∼103° for ODTS). This behavior indicated a clear shift toward hydrophobicity, with the ODTS’s longer alkyl chains (octadecyl) contributing to greater hydrophobicity compared to the OTS’s (octyl). Similarly, Janus-OTS and Janus-ODTS samples exhibited contact angles of ∼54° and ∼82°, respectively, which were lower than their isotropically coated counterparts. This aligned well with the TGA measurements showing a lower amount of hydrophobic silanes adsorbed on the particles due to their partial functionalization. Interestingly, the Janus-control sample displayed a contact angle of ∼37 °C, which was significantly greater than the TiO_2_-control sample. This observation confirmed the hypothesis that alkyl groups were still present on the titania surface after the washing step, resulting in effective Janus modification of titania nanoparticles by the waxes.

**Figure 5:**
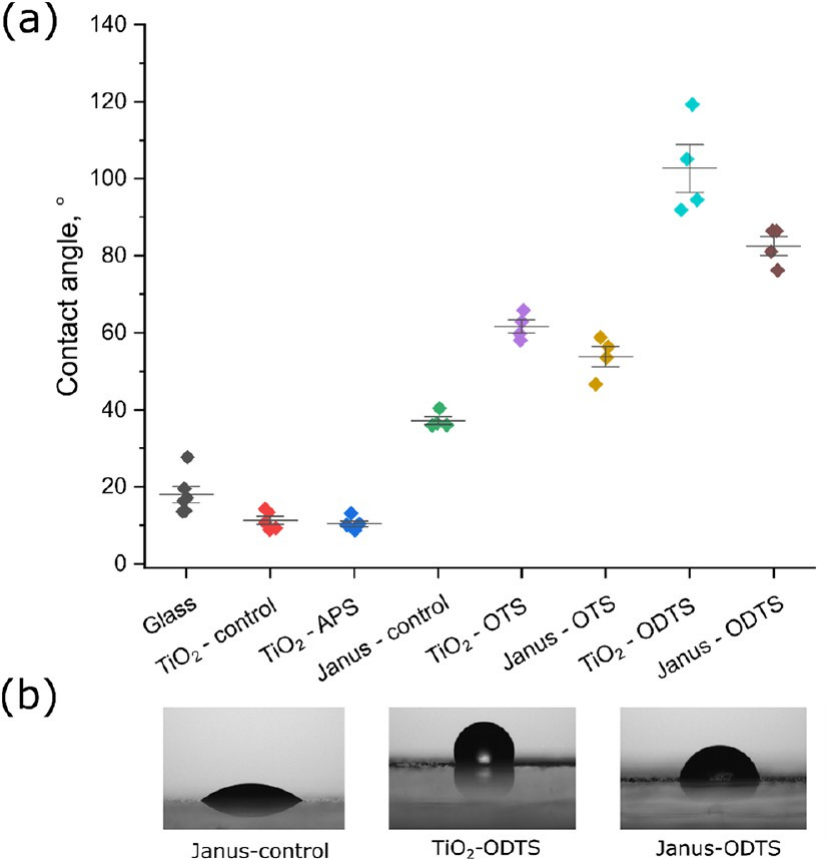
Contact angle values of the selected samples (a) and images of water droplets on ODTS-modified TiO_2_ and Janus-TiO_2_ surfaces (b).

The images below (Figure 5b) depict water droplets on ODTS-modified TiO_2_, Janus-ODTS and Janus-TiO_2_ surfaces. The TiO_2_–ODTS surface showed a highly hydrophobic behavior, corresponding to the contact angle of ∼103°. The Janus-TiO_2_ surface demonstrated a lower contact angle (∼75°) compared to the isotropic TiO_2_–ODTS. Finally, the Janus-control surface appeared much more hydrophilic with the contact angle of ∼35°. Additional droplet images for the tested samples are shown in Figure S3.

According to the classical Pickering emulsion theory, for the particles to adsorb and remain at the interface, their adsorption must reduce the system’s free energy according to eq 1.^26^

1 $$\Delta G=-\pi r^{2}\gamma_{\left\{\mathrm{ow}\right\}}\left(1-|\cos\theta |\right)$$

Where *r* is the particle radius and γ_{ow}_ is the oil–water interfacial tension and θ is the triphase contact angle between the particle, the water and the oil phase. From the equation, the energy reduction is highest for particles with θ = 90°, providing strong interfacial stabilization and resistance to detachment. While the thin film contact angle studies do not directly measure the triphase contact angle, the observed pattern provided insights into particle wettability and allowed predictions about their stabilization behavior at the oil–water interface. Therefore, the relationship between emulsion stabilization, the measured contact angle and the emulsion type will be established experimentally by analyzing the performance of particles in stabilizing emulsions.

### 3.5 APS Influence on Zeta Potential

Given that the contact angle measurements were better suited for the hydrophobic silane-modified titania, zeta potential (ZP) analysis was performed to characterize the change in dispersion stability due to the grafted amino groups from the APS. Figure 6 shows the variation of zeta potential with pH for TiO_2_ samples treated with different APS concentrations (0, 10, 100, and 1000 mM). Across all samples, the zeta potential decreased as the pH increased, transitioning from positive at low pH to negative at high pH. This dependency originated from the changes in the surface charge of titania, where the positively charged Ti–OH_2_^+^ groups were formed under the acidic conditions, while the negatively charged Ti–O^–^ groups dominated under the basic conditions due to the dissociation of the surface Ti–OH groups. In the absence of APS, the isoelectric point (IEP) was found to be ∼5.6, which was within the range of reported values in the literature.^27,28^ When titania was functionalized by the APS (100 and 1000 mM curves), higher positive zeta potentials at low pH and less negative zeta potentials at high pH were attained. This behavior was linked to the nature of the –NH_2_ groups, which are more basic (p*K*_a_ ∼ 9–10) than the −OH groups. In the range of pH 3–4, this difference did not significantly affect the degree of H^+^ association, but as the [H^+^] decreased, the differences in basicity became more pronounced effectively shifting the IEP value from 5.6 at 0 mM APS to 6.8 at 1000 mM APS. Once the pH became higher than the p*K*_a_ of APS, the amino groups were primarily deprotonated, which reduced the charge on the particles. In contrast, the unmodified titania under the same conditions could fully dissociate resulting in a higher negative charge density than the TiO_2_-APS formulations. Overall, these changes reflected the effectiveness of functionalization of the TiO_2_ surface by APS, where the amine groups introduced additional positive charges and shifted the IEP value by 1.2 units. In practice, the dispersions prepared at pH 7 exhibited completely different dispersibility with the 100 mM APS particles showing fast agglomeration (ZP = −1.98), while the 0 mM dispersion displayed good stability (ZP = −23.8). This pattern was found to be opposite at pH 5 in line with the reported measurements. At both pH 3 and 11, the minor changes in ZP did not substantially affect the hydrodynamic particle sizes, which were measured to be 131.6 ± 5.1 nm across all the formulations tested. Therefore, dispersions made under these conditions were chosen for the emulsification testing as the influence of hydrodynamic particle size in droplet stabilization could be fully eliminated.

**Figure 6:**
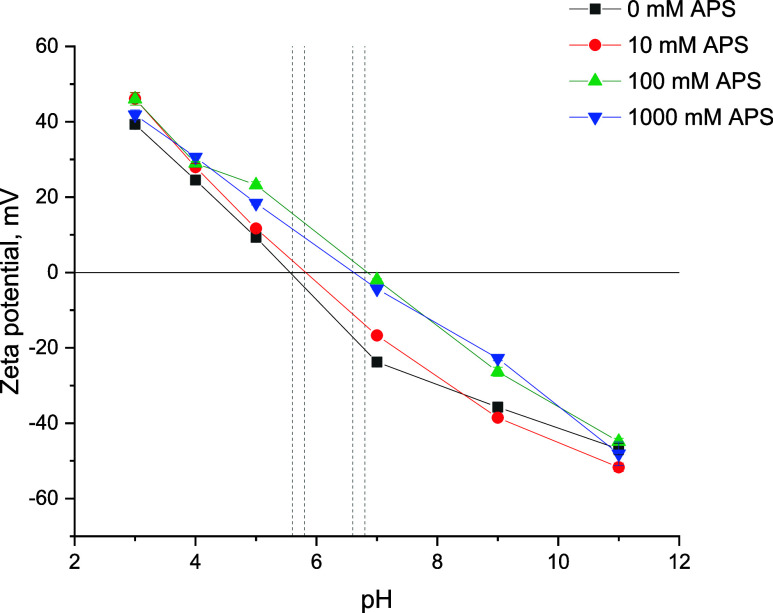
Variation of zeta potentials with respect to pH values for the TiO_2_-APS samples.

### 3.6 Emulsification Properties

#### 3.6.1 APS Functionalization

Having confirmed the successful modification of titania nanoparticles, the APS-functionalized TiO_2_ dispersions were used to make oil-in-water emulsions. At first, the emulsification by dispersions stabilized at pH 3 was tested with the corresponding droplet images depicted in Figure S4. In the absence of APS, no stable emulsions were formed as the water and oil phases immediately phase separated. However, the TiO_2_-APS particles could stabilize emulsions with the smallest droplet sizes achieved for the 100 mM APS dispersion (53.62 ± 10.49 μm). Still, most of the particles visually remained in the water phase, which was characteristic of highly hydrophilic particles as found by the contact angle measurements (Figure 5a). In contrast, a sharp reduction in the droplet sizes was noted when pH 11 dispersions were used (Figure S5). This phenomenon was observed even with the bare titania emulsions, which exhibited improved stability and the droplet sizes of 15.19 ± 5.36 μm. Based on the zeta potential dependency with respect to pH values (Figure 6), the difference in the absolute values of zeta potentials was too small to explain the observed phenomenon. Then, two oil/water control emulsions mixed at pH 3 and 11 revealed that pH 11 emulsions were inherently more stable even without any particles present. As a result, a negative charge on the droplets was attained, which provided repulsions between the droplets.

For the pH 11 emulsions prepared in the presence of particles (Figure 7, 0 h samples), slightly larger droplets were observed at 100 mM APS, with particles clearly visible on the surface of the droplets. However, at 1000 mM APS, the droplets became smaller compared to the 0 mM control (∼4.1 and ∼15.19 μm, respectively) and exhibited slight flocculation. Since the zeta potential was determined to be too high for the particle aggregation to take place (Figure 6), it was postulated that the particles had formed bridges between adjacent droplets due to electrostatic attraction between the negative oil and positive –NH_3_^+^ groups. This was backed by several works that have shown high efficiency of APS as a flocculant of negatively charged suspended colloids such as microalgae and humic substances.^29,30^ After 24 h, flocculation became even more pronounced in the 100 and 1000 mM samples, while the 0 and 10 mM samples remained well dispersed. On the other hand, more free oil at the top of the 0 and 10 mM samples was found, which indicated limited long-term stability.

**Figure 7:**
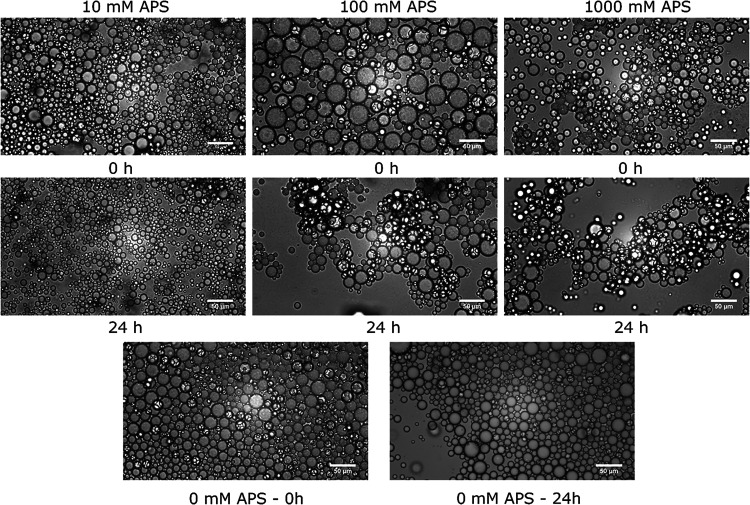
Microscope images of emulsions stabilized by TiO_2_-APS particles relative to the concentration of APS used in functionalization; comparison between the freshly mixed emulsions (0 h) and aged emulsions (24 h).

#### 3.6.2 OTS/ODTS Functionalization

More distinct differences were observed between the emulsions stabilized by hydrophobized titania. Figure 8 illustrates droplet distributions for both isotropic and Janus TiO_2_ particles with increasing hydrophobicity from left to right. The TiO_2_-control initially displayed uniformly sized, spherical droplets with relatively consistent distribution. Similarly, the Janus-control particle stabilized droplets were well-dispersed showing minimal clustering or flocculation. Regarding the TiO_2_–OTS sample, larger droplets, with significant coalescence were observed due to insufficient surface coverage as the particles were more difficult to disperse in the aqueous phase. In the same way, irregular sized droplets with micron-sized particles were noted with the Janus-OTS particles. Finally, the most hydrophobic, TiO_2_–ODTS, particles completely repelled water and immediately transferred to the oil phase without having produced a noticeable emulsion layer in the water phase. Therefore, only a dispersion of aggregated TiO_2_–ODTS particles in silicone oil is shown in the figure. In contrast, the Janus-ODTS particles could form emulsions made of very small droplets scattered around the aggregated particles. In summary, the microscope images demonstrated that the wettability of the particles identified by the contact angle studies (Figure 5) correlated well with the emulsion stabilization performance. For the OTS/ODTS-TiO_2_, poor stabilization of the emulsion droplets was noted as the produced particles were found to be highly oil-wetting. Still, the Janus-ODTS particles outperformed the ODTS-functionalized TiO_2_ in stabilizing the emulsions due to their higher hydrophilicity. In comparison, the hydrophilic TiO_2_-control and Janus-TiO_2_ particles produced emulsions with small, uniformly dispersed droplets, suggesting that the contact angle in the range of 10°–40° was essential for good dispersibility in the water phase and strong adsorption at the oil–water interface while preventing immediate droplet coalescence. The long-term stability of these emulsions will be evaluated in the next section.

**Figure 8:**
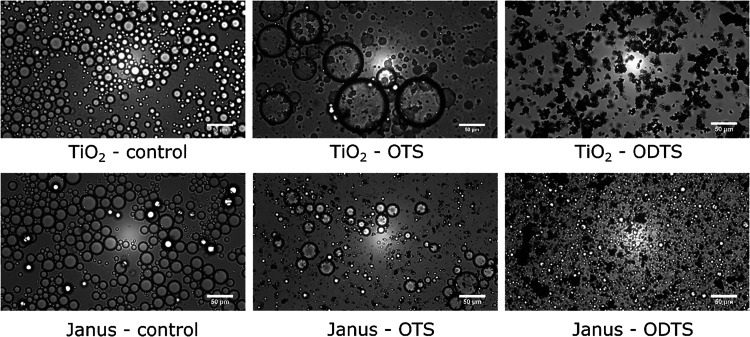
Microscope images of emulsions stabilized by OTS/ODTS functionalized TiO_2_ and Janus-TiO_2_ particles.

#### 3.6.3 Emulsion Stability

Long-term Pickering emulsion stability is critical for a variety of applications as it ensures consistent product performance and prevents phase separation that can compromise functionality and quality. Since the TiO_2_-APS emulsions were found to be stable within 24 h, an accelerated aging experiment by centrifugation was conducted. Panel (a) presents a bar graph that quantifies the amount of free oil separated from the emulsions after an accelerated aging process. This metric was directly related to the stability of the emulsions, where a lower amount of free oil indicated a higher stability and better droplet retention within the continuous phase. Based on the graph, a gradual increase in stability with respect to the APS concentration used in functionalization was observed. In the absence of APS, around 80% of the oil was collected after centrifugation, whereas the 100 mM formulation released less than 5% of the oil. This difference was attributed to the flocculation effect of APS, which bridged the droplets and reduced their mobility preventing them from coalescing. However, the 1000 mM formulation showed a slight increase in the amount of oil suggesting excessive flocculation that could have destabilized the system by bridging droplets too closely, potentially overcoming repulsive forces that prevented coalescence.

Since the emulsions stabilized by OTS/ODTS-modified titania exhibited different degrees of stabilization after mixing, a 60 day aging experiment was carried out. A photograph in Figure 9 b depicts the emulsions after aging. In the Janus-control sample, three layers could be identified, namely the top emulsion layer, the water phase and the sedimented particles. Although many particles have not been incorporated in the emulsion layer, the top layer showed a thick and dense layer indicating high stability. In contrast, almost no titania was observed in the water phase for the TiO_2_–OTS, Janus-ODTS and TiO_2_–ODTS samples, where titania was primarily dispersed in the oil phase. The Janus-OTS displayed a mixed behavior with both the particles and emulsion droplets were dispersed in the water phase. The TiO_2_-control was omitted due to rapid coalescence.

**Figure 9:**
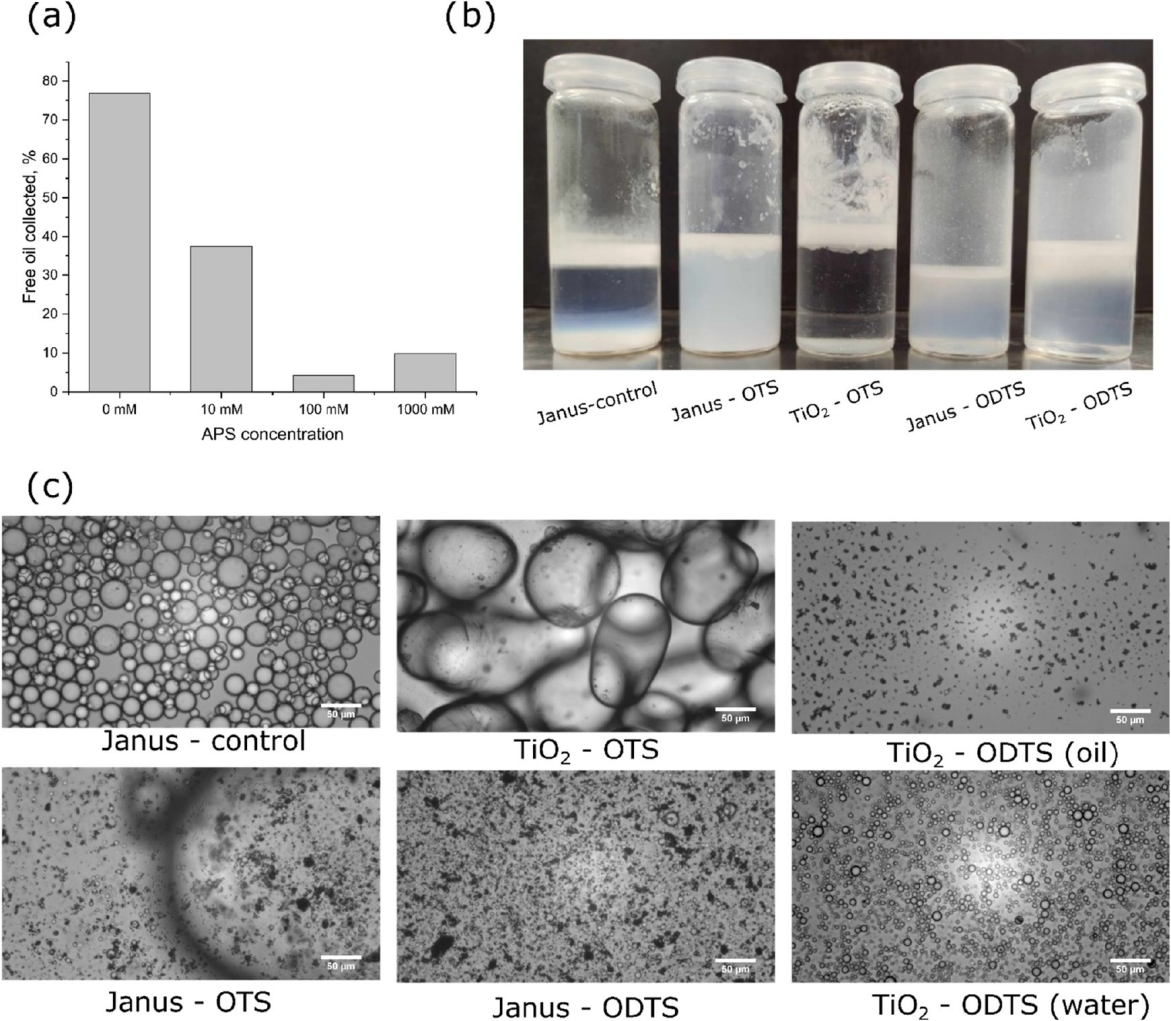
Amount of free oil collected after accelerated aging of TiO_2_-APS emulsions (a), photograph of the hydrophobized-titania stabilized emulsions after aging for 60 days (b) and their corresponding microscope images (c).

The corresponding microscope images of the droplets are shown in Figure 9c. For the TiO_2_–ODTS, microscope images of both the oil and the emulsion layers were separately shown in the figure with the dispersed particles in the oil phase and small droplets formed in the water phase. The resulting emulsion resembled the inverse Janus-control case, where the particles adsorbed to the oil–water interface from the oil phase rather than the water phase. Since the particles were highly hydrophobic (contact angle ∼100°), a clear separation between the phases was achieved. Small droplets in the emulsion layer were also observed in the Janus-ODTS emulsions. However, due to the unmodified −OH groups, the particles were partially dispersed in the water phase, resulting in a mixture of droplets and particles. The most critical change in droplet distribution was noted for the TiO_2_–OTS system, which underwent significant coalescence. This evolution was attributed to the lack of sufficient thickness of the hydrophobic layer around titania, which could have prevented droplets from coming into close contact and coalescing. Although ODTS was more hydrophobic, it possessed a longer alkyl chain (C18), which might have provided steric stabilization for the particles, although direct evidence for this effect is lacking. As a result, more uniform droplets could be produced during emulsification when using ODTS instead of OTS as a wetting agent. Coalescence was also noted for the Janus-OTS emulsion, although a higher concentration of free Janus-OTS particles was observed due to increased hydrophilicity. These findings revealed that intermediate particle wetting alone could not ensure high emulsion stability as the nature of the wetting agent influenced droplet stabilization mechanisms. In the case of APS, enhanced stability originated due to droplet flocculation and reduced mobility, whereas the ODTS provided a sufficiently thick steric hindrance layer. Excellent stability of the Janus-control emulsions was attributed to the combination of the intermediate wetting and long chain lengths, induced by the wax modification.

### 3.7 Photocatalytic Performance

The feasibility of TiO_2_-silane Pickering emulsions as photocatalytic systems was assessed by mixing the prepared emulsions with a 4-pb pollutant solution and monitoring their stability and the extent of photocatalytic degradation over time. Initially, laser diffraction analysis was employed to establish the initial droplet size distributions of the test emulsions (Figure 10a). It was evident that the surface modification by APS and Janus-route produced substantially smaller droplet sizes compared to the bare TiO_2_ stabilized emulsion. The reduction in size was associated with the higher concentration of particles fulfilling the criteria of intermediate wetting, which increased the affinity for the adsorption at the oil–water interface. The observed pattern was further supported by the visual appearance of the emulsions (Figure S6), showing high turbidity for the Janus-TiO_2_ and TiO_2_-APS emulsions, while the bare TiO_2_ emulsion demonstated creaming behavior and particle partitioning into the water phase.

**Figure 10:**
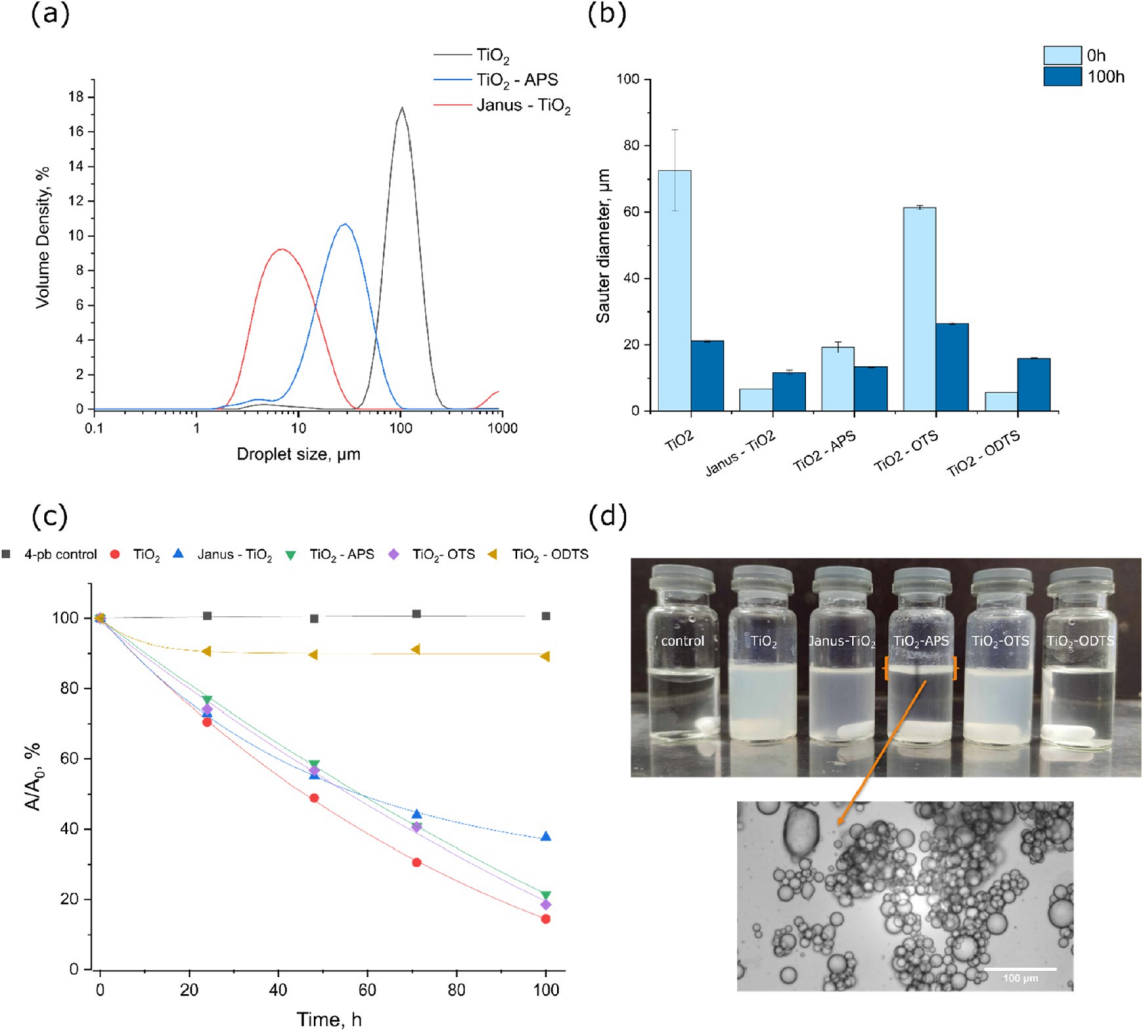
Volume distributions of the selected emulsions before the photodegradation experiments (a); variation in emulsion Sauter diameters before and after the photodegradation cycle (b); evolution of the 4-propyl benzoic acid peak absorbance under UV irradiation over time (c); digital photograph of the test samples after the photodegradation cycle (d, top); and optical microscopy image of the TiO_2_-APS emulsion after the photodegradation cycle (d, bottom).

During the photodegradation, varying degrees of coalescence were observed for all the emulsions, but the TiO_2_ and TiO_2_–OTS emulsions were found to be almost completely demulsified. This outcome is illustrated in panel d, where the demulsified samples appear light blue due to the Tyndall effect, characteristic of the dispersion samples. In contrast, the Janus-TiO_2_, TiO_2_-APS and TiO_2_–ODTS samples largely preserved their emulsion structure with only a slight shift in their Sauter diameters (Figure 10b). The reduction of Sauter diameters for the TiO_2_ and TiO_2_–OTS samples was explained by the coalescence of the large droplets, leaving only trace amounts of smaller droplets and aggregated particles in their test solutions. Due to the flocculation behavior by APS, the TiO_2_-APS emulsion formed a dense emulsion layer at the top of the sample allowing it to be collected and reused (Figure 10d). Despite the creaming and flocculation observed for the TiO_2_-APS emulsion, the individual emulsion droplets remained largely unaffected, providing small droplets and high surface area for photocatalysis. This was demonstrated by monitoring the evolution of 4-pb absorbance with respect to the irradiation time (Figure 10c), where the efficiency of TiO_2_-APS emulsion largely resembled that of the demulsified TiO_2_ and TiO_2_–OTS samples. In comparison, the Janus-TiO_2_ emulsion remained homogeneously dispersed, with a Sauter diameter of 12 μm, exhibiting minimal creaming and high turbidity. However, the high turbidity contributed to the reduction in the photocatalytic degradation rate due to light scattering. Despite the small droplet size, extremely low photocatalytic efficiency was noted for the TiO_2_–ODTS emulsion, attributed to the high affinity of the particles for the oil phase, where most TiO_2_–ODTS was dispersed (Figure 9c). To summarize, both Janus-TiO_2_ and TiO_2_-APS emulsions exhibited enhanced stability during the photodegradation with minimal reduction in the photocatalytic efficacy for the TiO_2_-APS system, offering a simple approach for photocatalyst reuse and recovery in wastewater treatment applications.

## 4 Conclusions

This study highlights the efficacy of organosilane-functionalized titanium dioxide (TiO_2_) nanoparticles in stabilizing Pickering emulsions and provides a comprehensive framework for tailoring nanoparticle surface properties to meet specific emulsification requirements. By employing organosilanes with varying hydrophilicity—APS, OTS, and ODTS—the wettability of TiO_2_ nanoparticles was systematically modified, enabling precise control over their emulsification behavior. Comprehensive analysis, including contact angle measurements, FT-IR, TGA, and zeta potential studies, confirmed the success of the functionalization process and its impact on nanoparticle properties.

APS-functionalized TiO_2_ nanoparticles were shown to significantly enhance emulsion stability by inducing droplet flocculation, a mechanism that contributed to prolonged stability in oil-in-water systems. Another key finding of this research was the determination of an optimal contact angle range (∼10–40°) for water on dispersion thin films, necessary for effective emulsification. Comparative studies of OTS- and ODTS-functionalized particles revealed the superiority of ODTS in stabilizing emulsions, owing to its longer alkyl chain that provided effective steric hindrance. This characteristic mitigated droplet coalescence more effectively than OTS, whose shorter chain length was insufficient for robust stabilization. Therefore, the particle wettability was not sufficient to determine the emulsification outcome, as the nature of the wetting agent also played a critical role in ensuring long-term stability.

Moreover, the development of Janus TiO_2_ particles through functionalization via CTAB facilitated uniform particle adsorption at the oil–water interface, resulting in homogeneous and well-dispersed titania-stabilized wax emulsions. The produced waxes allowed partial surface modification by the silanes, resulting in dual-wetting particles, which were found to be more hydrophilic than their isotropically coated counterparts. Furthermore, the Janus-control particles indicated superior stability and wetting properties resulting from CTAB adsorbed on the particles. Future studies employing high-resolution techniques such as HAADF-STEM with EDX mapping have been suggested to aid in the maturation and refinement of the synthetic approach.

The stability and photocatalytic performance of the emulsions were influenced by particle wetting and the initial emulsion structure. While the TiO_2_ and TiO_2_–OTS emulsions underwent significant coalescence and demulsification, Janus-TiO_2_ and TiO_2_-APS emulsions preserved their structure, allowing for photocatalyst reuse and recovery. Moreover, the TiO_2_-APS emulsion exhibited minimal loss in photocatalytic activity, making it a promising candidate system for wastewater treatment applications.

This work underscores the versatility of organosilane functionalization in tuning nanoparticle properties and demonstrates its potential for addressing challenges in a broad range of industrial and environmental applications, such as photocatalytic wastewater treatment.

## Supplementary Material



## Notes

Microscope image of molten Pickering wax emulsions; SEM image of solid waxes prepared by adsorbing CTAB on titania at pH 11; water droplet images on dispersion thin films for the remaining samples; TiO_2_-APS Pickering emulsions obtained at pH 3; summary of average droplet sizes for TiO2-APS emulsions prepared under different conditions (PDF)

## †

Novo Nordisk Pharmatech, Københavnsvej 216, Køge 4600, Denmark
