# Supplementary material for: Functionalization of TiO2 and Janus-TiO2 Nanoparticles with Organosilanes for Tunable Pickering Emulsification and Photocatalytic Wastewater Treatment
Source: ACS Omega. 2025 Sep 23;10(39):45608–20. doi: 10.1021/acsomega.5c05844 (PMC12508955; doi:10.1021/acsomega.5c05844)
Supplement: Supplementary file 1 [file ao5c05844_si_001.pdf]

# Supplementary Information for:

# Functionalization of $TiO_2$ and Janus-$TiO_2$ nanoparticles with organosilanes for tunable Pickering emulsification and photocatalytic wastewater treatment

Zygimantas Gricius and Gisle Øye*

* Corresponding author.

E-mail addresses: zygimantas.gricius@ntnu.no (Z. Gricius), gisle.oye@ntnu.no (G. Øye)

Ugelstad Laboratory, Department of Chemical Engineering, Norwegian University of Science and Technology (NTNU), 7491, Trondheim, Norway

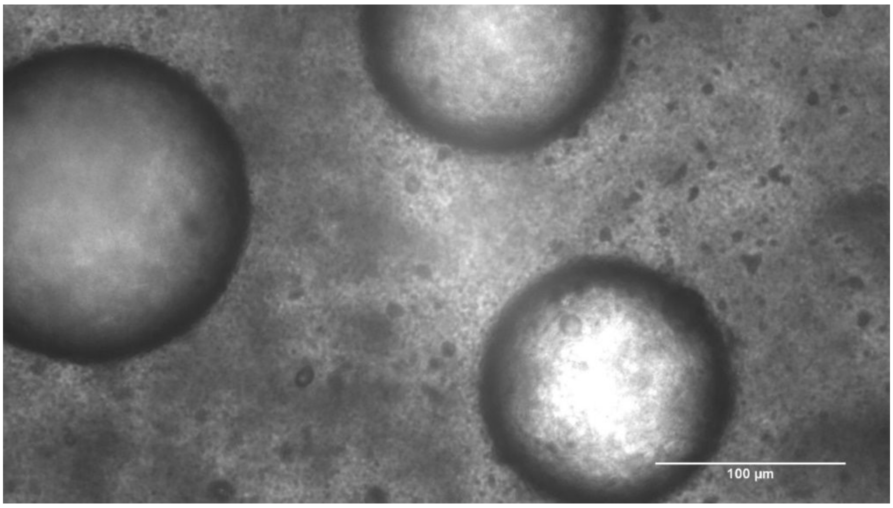


*Figure S1 Microscope image of molten Pickering wax emulsions*

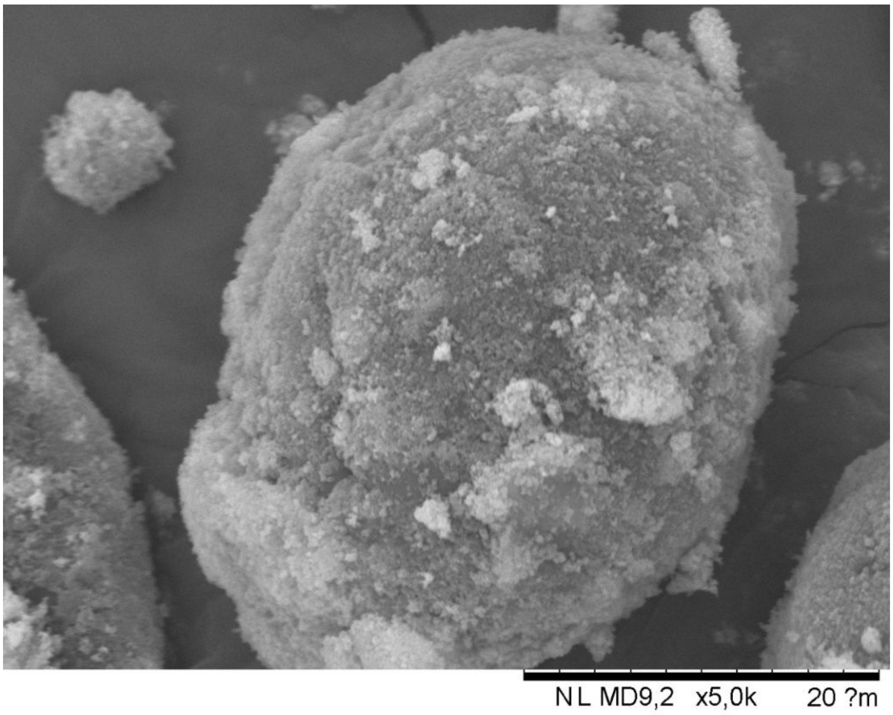


*Figure S2 SEM image of solid waxes prepared by adsorbing CTAB on titania at pH 11*

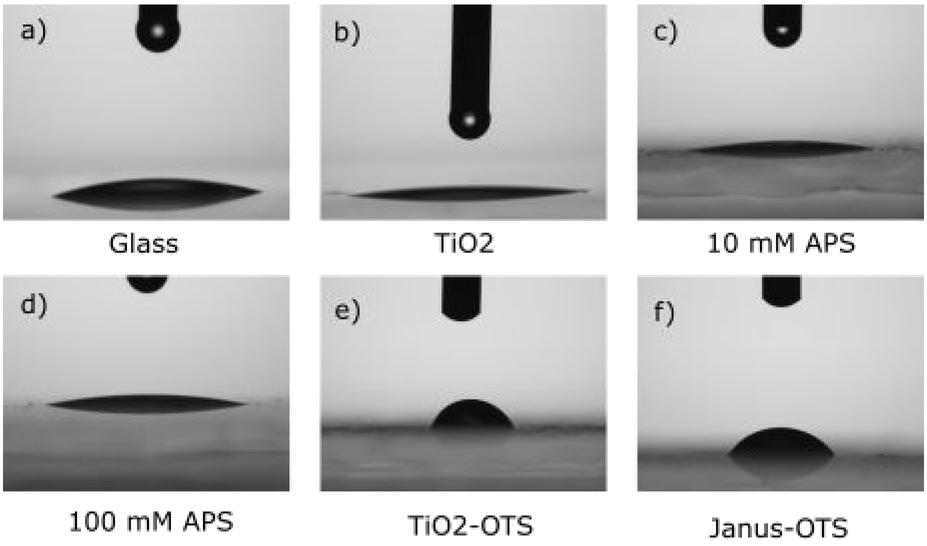


*Figure S3 Water droplet images on dispersion thin films for the remaining samples: bare glass substrate (a), spin-coated $TiO_2$ nanoparticles (NPs) on glass (b), spin-coated TiO2-APS NPs prepared at 10 mM (c) and 100 mM (d) APS concentrations, spin-coated TiO2-OTS NPs (e) and spin-coated Janus-$TiO_2$-OTS NPs (f)*

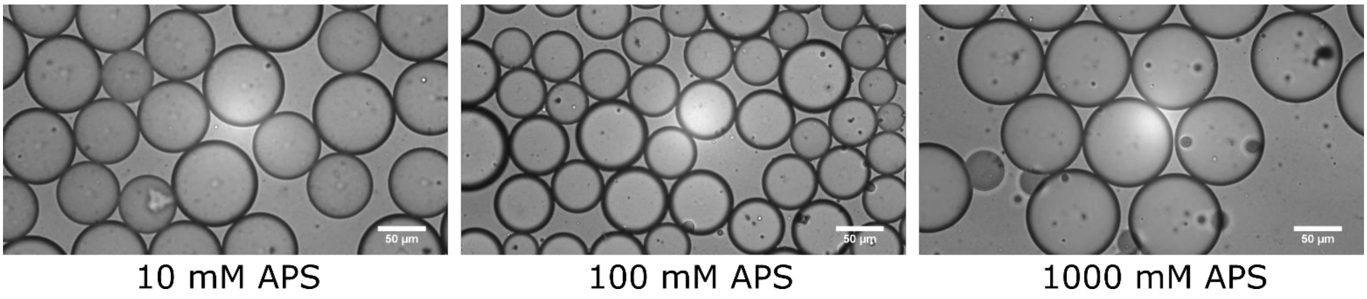


*Figure S4 $TiO_2$-APS Pickering emulsions obtained at pH 3*

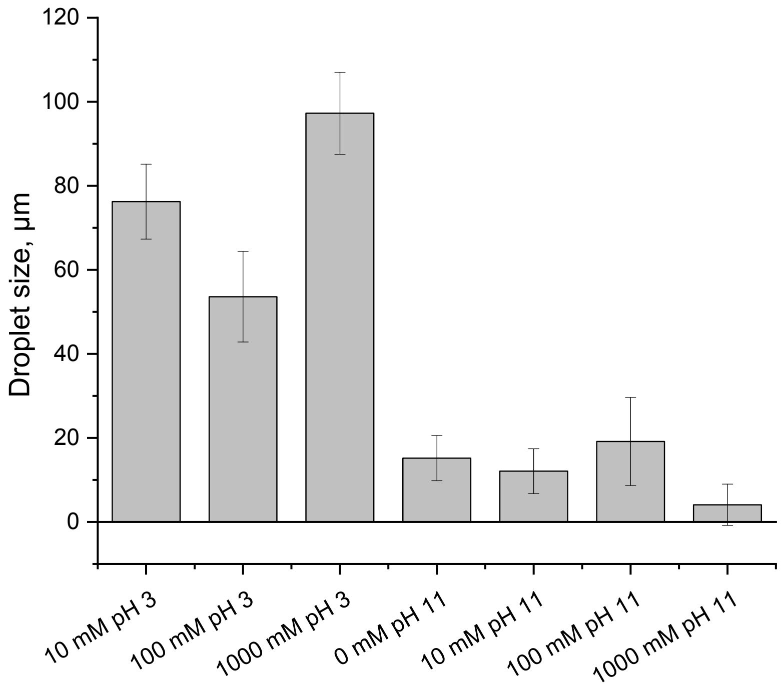


*Figure S5 Summary of average droplet sizes for $TiO_2$-APS emulsions prepared under different conditions.*

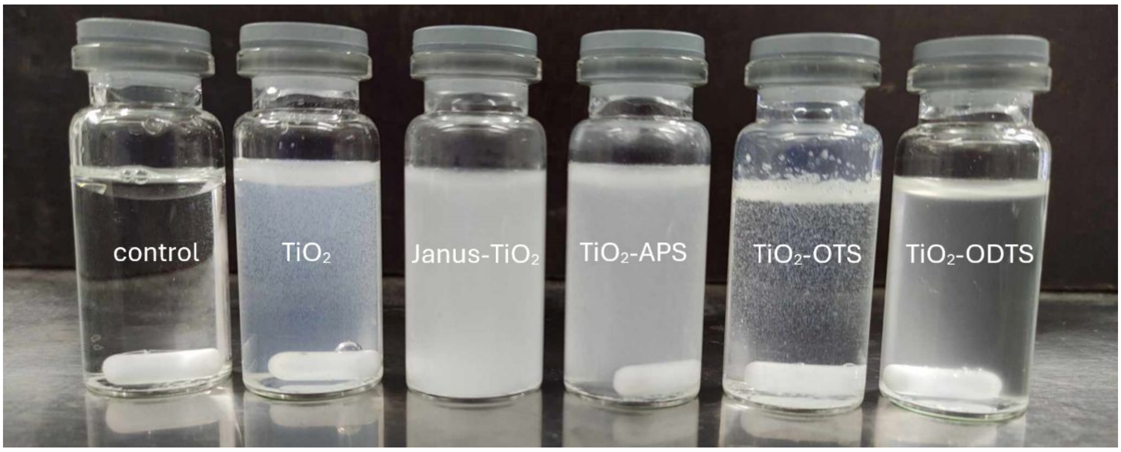


*Figure S6 Digital photograph of the test emulsions before the photodegradation cycle*
